# Clinical predictors of immediate response to a multimodal inpatient programme for chronic refractory musculoskeletal pain syndromes—a cross-sectional study

**DOI:** 10.1093/rap/rkaf024

**Published:** 2025-03-18

**Authors:** Tiffany Pretat, Thomas Hügle, Johanna Mettler, Marc Suter, Sandy Jean-Scherb, Pedro Ming-Azevedo

**Affiliations:** Department of Rheumatology, Lausanne University Hospital (CHUV), University of Lausanne, Lausanne, Switzerland; Department of Rheumatology, Lausanne University Hospital (CHUV), University of Lausanne, Lausanne, Switzerland; Department of Rheumatology, Lausanne University Hospital (CHUV), University of Lausanne, Lausanne, Switzerland; Pain Center, Department of Anesthesiology, Lausanne University Hospital (CHUV) and University of Lausanne, Lausanne, Switzerland; Pain Center, Department of Anesthesiology, Lausanne University Hospital (CHUV) and University of Lausanne, Lausanne, Switzerland; Department of Rheumatology, Lausanne University Hospital (CHUV), University of Lausanne, Lausanne, Switzerland

**Keywords:** chronic pain, musculoskeletal pain syndromes, multimodal programmes, prognostic factors

## Abstract

**Objectives:**

Chronic pain (CP) affects approximately 20% of the global population, leading to significant disability and economic burden. Multimodal programmes (MMPs) are the most effective short-term interventions for managing musculoskeletal chronic pain syndromes (MCPS). However, patient characteristics influence treatment response, requiring personalized approaches. This study aims to identify clinical, social and psycho-behavioural predictors of immediate response to a 2-week inpatient MMP for refractory MCPS.

**Methods:**

A cross-sectional study analysed 207 MCPS patients who completed an MMP at CHUV Lausanne, Switzerland, from March 2018 to November 2022. Validated questionnaires assessed pain severity, impact, kinesiophobia, catastrophizing and other factors before and after the programme. Univariate and multivariate analyses identified predictors of treatment response.

**Results:**

Significant improvements were observed in 9 out of 12 outcomes, including pain severity (*P* = 0.01), pain impact (*P* < 0.01), disability (*P* = 0.14), kinesiophobia (*P*<0.001) and catastrophizing (*P* < 0.001). Non-specific low-back pain, catastrophizing at entry, biomechanical disorders and psychiatric conditions were identified as key predictors of treatment response, respectively influencing 4, 3, 3 and 2 over 9 outcome measures in multivariable analysis. Non-specific low-back pain was linked to worse outcomes, whereas reductions in catastrophizing correlated with improved pain severity and kinesiophobia. Socioeconomic factors, such as disputes over disability financial aid, also influenced outcomes.

**Conclusion:**

This study confirms a modest yet significant immediate benefit of MMP for patients with refractory MCPS and provided a deeper insight into the predictors of treatment outcomes and their influence on various outcome measures. Further longitudinal studies are needed to confirm these findings and explore underlying mechanisms.

Key messages

- Significant improvement in 9 of 12 outcomes was observed for severe MCPS patients in this MMP.
- Non-specific low-back pain was associated with worse outcomes, while catastrophizing reduction improves pain severity and kinesiophobia.
- Socioeconomic factors, such as disputes over disability financial aid, also importantly influenced outcomes.

## Introduction

Chronic pain (CP) affects approximately 20% of the global population, leading to significant disability and economic burden [1]. In the USA, the annual costs of CP are estimated to range between $560 and $635 billion, with a substantial portion attributed to indirect costs such as lost productivity and disability [2].

Despite uncertainties about optimal treatment, MMPs has emerged as the most effective intervention, at least in short term [3–5]. Significant benefits of multicomponent treatment were documented in meta-analysis [4], and MMPs are proposed for the refractory cases in most guidelines [6]. However, CP is a heterogeneous group of diseases. The effectiveness of interventions depends on the underlying cause of pain and on patient’s characteristics. Such variability implies the necessity to adapt treatments to patients [6] and to select patients who are best suited to a particular approach.

Since 2018, the department of rheumatology of our institution (CHUV Lausanne, Switzerland) provides an MMP for patients with musculoskeletal chronic pain syndromes (MCPS) and fatigue syndromes. This 2-week inpatient programme includes the evaluations of patients by several health professionals, including rheumatologists, anesthesiologists, physiotherapists, occupational therapists, chiropractors, osteopaths and psychiatrists. The aim is to rediscuss the diagnoses, to build an overall action plan for the patient and to find solutions concerning health insurance-related issues. Evaluating the clinical and epidemiological factors associated with the response to this programme can both help selecting patients who benefit most from this kind of treatment and to better adapt the MMPs to the needs of patients. Such an analysis provides insights into how to assess and deal with the complexity involved in MCPS.

## Methods

### Study design

This is a cross-sectional study investigating clinical, social and psycho-behavioural characteristics of patients influencing the immediate response to our MMP, measured by the variability of a selection of questionnaires suitable for assessing the various dimensions involved in MCPS. This study conforms to the Strengthening the Reporting of Observational studies in Epidemiology (STROBE) guidelines for this research area [7, 8].

### Setting and participants

Data were collected retrospectively from the electronic medical records of all 207 consecutive patients’ refractory musculoskeletal pain syndromes who participated in our 2-week inpatient MMP between March 2018 (inauguration of the MMP) and November 2022. Five patients were excluded after not completing the programme. The inclusion criteria for the MMP are MCPS, failure of ambulatory care, ≥ 18 years of age and the ability to fluently understand and speak the French language. Inclusion criteria specific for the study also include the agreement to the informed consent. Exclusion criteria for the MMP were the existence of cognitive, physical, cultural or psychiatric difficulties that prevent patients from understanding and/or adhering to MMP procedures. Ethics approval for performing this study on the dataset was granted by Commission cantonale d'éthique de la recherche sur l’être humain (CER-VD), on 6 December 2021. Project-ID 2021–00853.

## Variables

### Outcome measures

Patients were asked to complete validated French versions of specific self-report questionnaires before and immediately after the MMP. The questionnaires used were chosen by a group of physician specialists in the treatment of MCPS beforehand, when designing the MMP. They are the validated and traditionally used French versions for patients with CPS and assess various aspects of this condition, including cognitive behavioural factors: kinesiophobia (Tampa Scale of Kinesiophobia, TSK), catastrophism (Pain Catastrophizing Scale, PCS), avoidance beliefs regarding work and physical activity (Fear-Avoidance Beliefs Questionnaire for physical activities, FABQ-P, and work, FABQ-W) and patterns of activities (Patterns of Activity Measures for overdoing, POAM-O, avoiding, POAM-A, and pacing, POAM-P). They also evaluated emotional distress (anxiety and depression using the Hospital Anxiety and Depression Scale, HAS and HDS), physical functioning (Brief Pain Inventory impact, BPI-I, and Oswestry Disability Index, ODI) and pain severity (BPI-S). The difference in scores before and after the programme, termed ‘Delta’ or ‘Δ’ questionnaires, was used as measure of programme’s immediate outcome, and the ΔBPI impact was the programme’s primary outcome measure and all other were secondary ones. The questionnaires were completed online using secure software (Redcap^®^), and patients’ identities were replaced by a security code. Access to questionnaires is sent to patients 3 days before hospitalization. Patients who fail filling up the electronic forms alone do it with assistance at entry and at depart.

Kinesiophobia: the TSK is a psychological assessment tool used to measure kinesiophobia, or fear of movement, through 17 items that gauge participants’ feelings about pain and movement. Commonly used in clinical settings, the TSK helps determine how fear influences a patient’s avoidance of physical activity, guiding treatment strategies and interventions [9].

Catastrophism: the PCS is a psychological tool used to measure an individual’s tendency to catastrophize pain, a process where pain is anticipated or experienced with exaggerated negative mental responses. The PCS evaluates thoughts and feelings about pain across three dimensions: rumination, magnification and helplessness. Widely utilized in both clinical and research settings, the PCS helps assess the impact of pain catastrophizing on pain experiences and outcomes, especially in managing CP [10].

Avoidance beliefs regarding work and physical activity: the FABQ is a tool designed to evaluate individuals’ beliefs about how physical activity and work might cause or worsen pain. It is frequently used to understand how these beliefs contribute to the avoidance of physical activity and the resulting disability in CP conditions [11].

Patterns of activities: the POAM questionnaire is designed to evaluate daily activity behaviours in individuals with CP. It identifies three main behavioural strategies: pacing (POAM-P), which is adaptive, and avoidance (POAM-A) and overdoing (POAM-O), which are often maladaptive and linked to the development and persistence of CP. POAM-A measures the extent to which individuals avoid activities due to fear of pain or re-injury. POAM-O assesses how individuals exceed their physical limits during activities, often ignoring pain signals, which may lead to increased pain and a cycle of overactivity followed by enforced rest [12].

Anxiety and depression: the Hospital Anxiety and Depression Scale (HAS and HDS respectively) is a common screening tool used to detect potential anxiety disorders and depression. It is especially valuable for assessing patients suffering from ongoing physical discomfort, such as MCPS [13].

Evaluation of pain severity, physical functioning and quality of life: the BPI is a standardized tool often used to measure pain severity and its effects on daily activities in individuals with CP. The BPI-Severity (BPI-S) section has patients rate their pain from 0 (no pain) to 10 (worst pain imaginable), while the BPI-Impact (BPI-I) section assesses how pain affects aspects of daily life such as general activity, mood, mobility, work, social interactions, sleep and enjoyment of life. In the study’s longitudinal component, not discussed in this paper, BPI-Impact variation was selected as the primary outcome for MMPs, with other questionnaires serving as secondary measures [14].

### Clinical and epidemiological variables

In a consensus of experts, we have previously chosen a list of candidate clinical and epidemiological variables believed to be worth investigated and defined each of these variables as objectively as possible aiming reducing bias [15]. The epidemiological variables evaluated were age, gender, origin, origin of parents, marital status, paternity, living alone, type of work, time away from work and request for financial assistance related to disabilities. The general clinical variables assessed were body mass index (BMI), present or past clinical hypermobility, main clinical comorbidities (diabetes, dyslipidaemia, sleep apnea, smoking), menopause and C-reactive protein (CRP) and sedimentation rate (SR). The variables specific for pain included the site of pain (spinal, peripheral, diffuse), the main structures affected (joints, tendons, entheses, bursa, nerve roots, peripheral nerves), type of pain (nociceptive, nociplastic or neuropathic), presumed origin of pain (degenerative, biomechanical, inflammatory, microcrystalline, nociplastic, functional), meeting American College of Rheumatology’s 2010 and Fibromyalgia Rapid Screening Test (FiRST) criteria for fibromyalgia, presence of hyperalgesia and allodynia objectified by Algopeg^®^, history of skeletal surgeries, presence and type of autoimmune diseases associated with pain, history of pain in childhood and adolescence and sleep analysis by actigraphy. The psychiatric variables assessed included depression, anxiety, diagnosis of personality disorders, bipolar disorder, post-traumatic stress disorder (PTSD) or enduring personality change after catastrophic experience (EPCACE), alexithymia, history of insecurity or neglect in childhood and adolescence. The medications assessed included opioid and non-opioid analgesics, anti-inflammatories, antidepressants, anticonvulsants, benzodiazepines, Z-drugs, neuroleptics, myorelaxants, prednisone and immunosuppressants. Several variables were assessed by different methods as detailed elsewhere [15]. BMI, for example, was tested as a numerical variable, as a multi-categorical variable or as a binary variable (according to World Health Organization [WHO] definitions). The above-mentioned scores’ results at entry were also tested as clinical variables. For instance, patients’ score on PCS **at entry** (**En**PCS) was tested as a clinical variable for catastrophism.

### Statistical analysis

Evolution of scores from baseline to discharge: scores were compared pre- to post-treatment with Wilcoxon test for non-parametric distribution.

#### Univariate analysis

In the univariable analysis, 153 clinical or demographical variables and variation (before and after MMP) of the 12 above-cited questionnaires were tested. We used Pearson’s test to compare two numerical variables, the Spearman test to compare two categorical variables and the point-biserial test to compare two numerical and categorical variables.

#### Multivariate analysis

*Variables pre-treatment*. All variables associated with score variance with a 95% confidence interval were used in the multivariate analysis. In addition, all variables associated with quantitative change in scores with 80% confidence interval (*P*-value of ≤ 0.2) were also tested in the multivariable analysis [16]. Due to the ambiguous distribution of Δscores, non-parametric Kruskal–Wallis tests were preferred for categorical independent variables. For comparison with independent numeric variables, we used the Spearman’s rank correlation.

To optimize the validity of the multivariate models, variables limiting the number of observations due to missing data were excluded from the analysis. To obtain enough observations, some compatible variables were combined. Notably, PTSDs were combined with enduring personality changes after catastrophic experience (EPCACE), benzodiazepine use was combined with Z-drugs use, antiseizure medications (ASM) use was combined with GABAergic, and prednisone was combined with biologic drugs use. Additionally, strong and weak opiates were gathered. If variables were collinear, only the one considered to be the most relevant would be included in the model. Variables aiming at describing the sample but considered to have limited clinical relevance were excluded before backward elimination (e.g. ‘buttock pain’, ‘vertebral pain’, ‘spondylar pain’). Binary variables were required to perform multivariate regressions. If variables presented more than two categories, an effort was made to transform them into binary without losing information. In the end, the total number of independent variables before backward elimination was comprised between 6 and 15.

#### Multiple linear regressions

A backward elimination process was used, until every independent *P*-values attained a threshold ≤ 0.05. Presence of outliers, linearity, multicollinearity, normality of the residuals and homoscedasticity was assessed to guarantee the quality of the models. No outliers were found, and models globally respected linearity, multicollinearity and homoscedasticity.

The normality of the residuals was not always respected (ΔBPI-S, BPI-I, FABQ-P, PCS), but models with large samples tend to be considered robust even when normality is violated (see Discussion section). To confirm our results, a parallel statistical analysis was performed with categorized scores: the outcome was either score improvement or no improvement/worsen score. Multiple logistic regression with backward elimination revealed partial congruence with the multiple linear regressions. Since categorizing holds a risk of information loss, multiple linear regressions were preferred.

*Post-hoc analysis*. To contextualize the models, post-hoc univariate analyses were performed on multiple relevant variables, including each significant variable of the BPI-I model (main outcome) and some variables of the ODI model. The model variables were compared with demographic, clinical and psychiatric characteristics, medication, sleep analysis and score evolution. Chi-square tests were used to compare categories, Student’s *t*-test was used to compare parametric distributions across two groups, and Mann–Whitney U tests (MW-U) were used when distributions were non-parametric. The direction of associations was assessed with box plots. Wilcoxon tests were used to compare pre- and post-treatments scores for general anxiety disorder (GAD) and personality disorders in the ODI model.

Data were analysed by the investigators with Excel office 2019 and Stata/MP 13 and are available at https://doi.org/10.5281/zenodo.14912373.

## Results

### Demography and clinical features

Two hundred and seven (207) patients initially participated in the study, but five were excluded—three for not completing the MMP and two for not completing the questionnaires—leaving a final sample of 202 participants. The majority (73.3%) of the 202 participants were female with an average age of 47 years, plus or minus 10 years. The median body mass index (BMI) for the group was 27, with a range spanning from 16.3 to 50.3. Geographically, the composition of the sample was quite diverse: 44.1% of the participants were Swiss, 39.6% were from other European countries, 10.5% were non-Europeans, and the origins of 5.9% are unknown. In terms of seeking financial assistance due to disability, 68.7% of the patients have pursued disability financial aid (DFA), while 45.5% are awaiting a response regarding DFA. Additionally, 13.4% have already received DFA, whereas 8.4% were refused such aid. Regarding clinical characteristics, back pain was the most common site of pain, affecting 89.6% of the sample, and the predominant type of pain was nociplastic, experienced by 73.8% of the patients. Concomitant fibromyalgia was present in 78% of the group, and 22% suffer from immune-mediated disorders. Psychiatric comorbidities were also notable, with 74% of the sample having some form of psychiatric condition. Among these, depression was the most common, affecting 56% of the patients. Furthermore, 15.4% have been diagnosed with enduring personality change after a catastrophic experience (EPCACE), and 9.9% suffer from PTSD. The complete sample description of demographic and clinical characteristics, medication and sleep disturbances can be found in Prétat *et al.*[15].

### Evolution of scores from baseline to discharge

We observed statically significant improvements (negative Δ-scores) after the 2 weeks of MMP in 9 of the 12 evaluated scores, including measures of pain severity and impact, pain-related believes, kinesiophobia, catastrophizing, depression and disability (Table 1). No effect was observed on FABQ-work and POAM for overdoing and avoidance.

**Table 1.** Evolution of outcome measures during the 14-day inpatient multimodal programme

	Entry *N* = 197	Discharge *N* = 172	Δ: ‘ - ’ mean improvement	
Scores Interpretation	Median, [Range], [25–75th percentile]	Median, [Range], [25–75th percentile]	Median, [Range], [25–75th percentile]	*P*-value
Pain severity (BPI-S) 0–10: no predefined cutoffs	6.25, [0; 10], [5; 7.5]	6, [0; 10], [4.5, 7.3]	−0.3, [−7.5; 2.5], [−1.3; 0.5]	**0.010**
Pain impact (BPI-I) 0–10: no predefined cutoffs	6.6, [0; 10], [4.9; 7.9]	5.6, [0; 9.1], [3.6; 6.9]	−0.7, [−7.3; 6.4], [−2.1; 0.3]	**<0.001**
Back pain disability (ODI) 0–20: minimal; 21–40: moderate; 41–60: severe; 61–80: disabling; 81–100: bedridden	48, [12; 82], [40; 58]	46, [6; 78], [36; 56]	−2, [−56; 28], [−8; 4]	**0.014**
Fear-avoidance beliefs on physical activities (FABQ-P) 0–24: poor prognosis if > 15	18, [0; 24], [13; 22]	14, [0; 24], [9; 19]	−2, [−20; 18], [−7; 1]	**<0.001**
Fear-avoidance beliefs on work activities (FABQ-W) 0–42: poor prognosis if > 34	19, [0; 42], [0; 33]	21, [0; 42], [0; 35]	0, [−42; 42], [−5; 8]	0.193
Pattern of activity-Overdoing (POAM-O) 0–40: no predefined cutoffs	24, [0; 40], [16; 30]	23, [0; 40], [17; 30]	0, [−25; 36], [−6; 6]	0.781
Pattern of activity-Pacing (POAM-P) 0–40: no predefined cutoffs	29, [0; 40], [18; 36]	30, [0; 40], [21; 37]	1, [−27; 35], [−3; 7]	**0.010**
Pattern of activity-Avoidance (POAM-A) 0–40: no predefined cutoffs	30, [0; 40], [23; 36]	28, [0; 40], [22; 36]	0, [−36; 36], [−6; 4]	0.135
Anxiety (HAS) 0–7: normal; 8–10: borderline; 11–21: abnormal	12, [1; 21], [9; 15]	10, [1; 21], [6; 14]	−1, [−11; 13], [−3; 1]	**<0.001**
Depression (HDS) 0–7: normal; 8–10: borderline; 11–21: abnormal	9, [1; 21], [6; 13]	8, [1; 21], [5; 12]	−1, [−10; 8], [−3; 1]	**0.008**
Catastrophism (PCS) risk if > 20; abnormal if > 30	32, [0; 52], [21; 40]	26, [0; 52], [15; 36]	−4, [−31; 33], [−10; 2]	**<0.001**
Kinesiophobia (TSK) 0–51: unlikely; 52–60: possible; 61–100: likely	46, [23; 64], [39; 52]	43, [21; 68], [36; 49]	−2, [−26; 31], [−6; 2]	**<0.001**

BPI: Brief Pain Inventory; FABQ: Fear-Avoidance Beliefs Questionnaire; HAS and HDS: the Hospital Anxiety and Depression Scale; ODI: Oswestry Disability Index; PCS: Pain Catastrophizing Scale; POAM: Patterns of Activity Measures; TSK: Tampa Scale of Kinesiophobia.

Bold indicates statistically significant.

We evaluated the correlations between clinical and/or demographic variables with the variation in questionnaire scores before and after hospitalization (delta, or Δ-scores). In the univariable analysis, 17 clinical and/or demographic variables significantly correlated with score variation (*P* < 0.05). The multivariable analysis included these 17 variables and 38 others that obtained *P* < 0.2 in the univariable analysis. A total of 26 variables obtained significant multivariable correlations with different Δ-scores (Table 2).

**Table 2.** Clinical and demographical variables with significant negative or positive correlation to scores modification after the multimodal programme (Δ-scores) in univariate and multivariate analysis

Delta (Δ) scores	**Variable association in univariate analysis.** *P*-values in **bold** indicate *P*-value ≤ 0.05; otherwise, *P*-value ≤ 0.2 (suitable to be tested in multivariate analysis)	Multivariate analysis	*N* (%)	Beta^a^, *P*, [95% CI]	**Model** *n*, *P*, *R*^2^ adjusted
ΔPain impact (ΔBPI-I)	**Non-specific low-back pain** (*P* = 0.044), **peripheral OA** (*P* = 0.008), radiculopathy, relationship status, any back pain, cervical pain, sleep inefficiency, depression, personality disorder, TCA, trazodone, myorelaxants, EnBPI-S, EnFABQ-W, EnPOAM-O, EnPCS	Non-specific low-back pain, Peripheral OA, Trazodone	131 (64.9) 57 (28.2) 23 (11.4)	0.155; **0.038**; [0.042; 1.43] 0.186; **0.013**; [0.020; 1.67] −0.177; **0.018**; [−2.41; −0.23]	171 **0.002** 0.071
ΔPain severity (ΔBPI-S)	**Non-specific low-back pain** (*P* = 0.041), **PTSD and EPCACE** (*P* = 0.011), age, gender, DFA, time off work, any back pain, biomechanical disorder, autoimmune rheumatism at MMP entry, AD, BZD and Z-drugs, myorelaxants, EnFABQ-P, EnBPI-I, EnHAS, EnPCS	DFA, Any back pain, Biomechanical disorder, PTSD+EPCAC, Myorelaxants, EnPCS, EnFABQ-P	136 (68.7) 182 (90.1) 68 (33.7) 34 (16.8) 28 (13.8) 195 (96.5) 195 (96.5)	0.218; **0.003**; [0.25; 1.20] 0.197; **0.007**; [0.27; 1.66] −0.175; **0.015**; [−1.08; −1.12] −0.240; **0.001;** [−1.60; −0.43] −0.193; **0.007**; [−1.45; −0.23] −0.283; **0.000** [−0.06; −0.02] 0.171; **0.030 [**0.00; 0.08]	167 **0.000** 0.195
ΔBack-pain handicap (ΔODI)	**Relationship status** (*P* = 0.046), **any back pain** (*P* = 0.012), **non-specific low-back pain** (*P* = 0.001), **personality disorder** (*P* = 0.009), **EnHAS** (*P* = 0.018), **EnHDS (***P*** = **0.048), **EnPCS (***P* = 0.040), **EnTSK** (*P* = 0.002), nociplastic pain, enthesopathy, GAD, opiates, sleep efficiency, CRP, gender, hypermobility, EnPOAM-O, EnPOAM-A	Non-specific low-back pain, Personality disorder, EnTSK, Opiates binary	131 (64.9) 54 (26.7) 195 (96.5) 202 (100)	0.270; **0.000**; [2.89; 9.22] 0.152; **0.035**; [0.27; 7.13] −0.252; **0.001**; [−0.49; −0.14] 0.147; 0.041; [0.13; 6.34]	168 **0.000** 0.159
ΔFear-avoidance beliefs-Physical (ΔFABQ-P)	**Gender** (*P* = 0.044), **biomechanical disorder** (*P* = 0.019), **confirmed autoimmune rheumatism** (*P* = 0.036), binary BMI, DFA, parenthood, cervical pain, shoulder pain, nociceptive pain, enthesopathy, AD, myorelaxants, paracetamol/metamizole, EnFABQ-W, EnHAS, EnPCS	Gender (male), Cervical pain, Biomechanical disorder, EnHAS	54 (26.7) 136 (67.3) 68 (33.7) 195 (96.5)	0.169; **0.025**; [0.32; 4.80] 0.203; **0.007**; [0.78; 4.93] −0.164; **0.029**; [−4.53; −0.24] −0.168; **0.026**; [−0.44; −0.03]	171 **0.001** 0.084
ΔFear-avoidance beliefs-Work (ΔFABQ-W)	**Other psychiatric conditions** (*P =* 0.049), any back pain, lumbar pain, non-specific low-back pain, shoulder pain, peripheral neuropathy, abnormal Algopeg^®^, dual antidepressants, TCA, GAD, EPCACE, alexithymia, pain in childhood, binary BMI, EnBPI-S, EnPOAM-A	Alexithymia	69 (34.2)	−0.162; 0.035; [–12.15; –0.46]	171 **0.035** 0.026
ΔCatastrophism (ΔPCS)	**Non-specific low-back pain** (*P* = 0.005), **TCA** (*P* = 0.010), biomechanical disorder, nociplastic pain, BZD and Z-drugs, NSAID, age, sleep fragmentation, sleep efficiency, CRP, EnFABQ-W, EnHAS	Non-specific low-back pain, Biomechanical disorder, TCA, BZD and Z-drugs, Sleep efficiency	131 (64.9) 68 (33.7) 22 (10.9) 71 (35.2) 85 (42.1)	0.183; **0.016**; [0.73; 6.99] −0.158; **0.038**; [−6.79; −0.20] 0.221; **0.004**; [2.22; 11.53] −0.160; **0.036**; [−6.58; −0.22] −0.157; **0.04**; [−33.74; −0.88]	159 **0.000** 0.111
ΔKinesiophobia (ΔTSK)	**Time off work** (*P* = 0.001), **personality disorder** (*P* = 0.013), **PTSD and EPCACE** (*P* = 0.010), **BZD and Z-drugs** (*P* = 0.033), DFA, parenthood, radiculopathy, biomechanical disorder, TCA, trazodone, NSAID, myorelaxants, EnHAS, EnPCS	Radiculopathy, BZD and Z-drugs, Personality disorder, Time off work, EnPCS	34 (16.8) 71 (35.2) 54 (26.7) 192 (95) 195 (96.5)	0.266; **0.001**; [1.88; 7.67] −0.143; **0.046** [−4.50; −0.04] 0.240; **0.003** [1.17; 5.83] 0.207; **0.004** [0.11; 0.53] −0.303; **0.007** [−0.21; −0.03]	163 **0.000** 0.184
ΔPattern of activity-Avoiding (ΔPOAM-A)	**Fragmented sleep** (*P* 0.038), **EnHAS** (*P* = 0.026), age, relationship status, hyperlaxity, PTSD and EPCACE, other psychiatric conditions, AD, BZD and Z-drugs paracetamol/metamizole, immunosuppressants, EnBPI-I, EnFABQ-P	EnHAS, EnFABQ-P, BZD and Z-drugs	195 (96.5) 195 (96.5) 71 (35.2)	0.251; **0.002**; [0.17; 0.74] −0.177; **0.025**; [−0.49; −0.03] −0.155; **0.041**; [−5.59; −0.12]	171 **0.002** 0.083
ΔPattern of activity-Overdoing (ΔPOAM-O)	**Hyperlaxity** (*P* 0.002), **EnTSK** (*P* = 0.045), age, conflict zone, DFA conflict, shoulder pain, nociceptive pain, biomechanical disorder, depression, trazodone, immunosuppressants, EnBPI-I, EnPCS	Age, DFA conflict, Hyperlaxity, EnBPI-I, EnPCS	- 109 (55.1) 50 (27.6) 195 (96.5) 195 (96.5)	−0.223; 0.006; [−0.36; −0.06] 0.163; 0.043; [0.10; 6.22] −0.208; 0.011; [−8.10; −1.08] −0.225; **0.015**; [−1.81; −0.20] 0.233; **0.017** [0.03; 0.33]	149 **0.000** 0.141

The beta coefficients indicate the direction of the association. A (-) coefficient indicates an improvement. All multivariate models were statistically robust. ‘En’ before scores’ acronym stands for ‘Entry’, meaning the score’s results at entry, which were tested as clinical variable. Scores: Brief Pain Inventory (BPI), Oswestry Disability Index (ODI), Fear-Avoidance Beliefs Questionnaire (FABQ), Pain Catastrophizing Scale (PCS), Tampa Scale of Kinesiophobia (TSK), Patterns of Activity Measures (POAM) Avoiding (POAM-A) and Overdoing (POAM-O), and the Hospital Anxiety and Depression Scale (HAS and HDS).

AD: antidepressants; BMI: body mass index; BZD: benzodiazepines; CRP: C-reactive protein; DFA: disability financial aid; EPCACE: enduring personality change after catastrophic experience; GAD: general anxiety disorder; MMP: multimodal programme; NSAID: non-steroidal anti-inflammatory drugs; OA: osteoarthritis; PTSD: post-traumatic stress disorder; TCA: tricyclics and tetracyclic antidepressants.

Of the 15 significant variables in the univariable analysis, 4 were associated with more than 1 Δ-score. These included non-specific low-back pain, which was negatively associated with improvement in pain impact (BPI-I), pain severity (BPI-S), handicap associated with back pain (ODI) and catastrophism (PCS). Similarly, 6 of the 23 variables associated with score variation in the multivariable analysis influenced more than one score. These included non-specific low-back pain and biomechanical disorders, both of which influenced three scores each. Age, the use of benzodiazepines and Z-drugs, receiving DFA and bearing personality disorders influenced two different questionnaires each (Table 3).

**Table 3.** Variables which more often influenced scores’ variation (Δ-scores) in univariable and multivariable analysis

Clinical variables	Outcomes measures (Δ-scores)									
	ΔCatastrophism (PCS)	ΔPain impact (BPI-I)	ΔPain severity (BPI-S)	ΔBack-pain handicap (ODI)	ΔFear-avoidance beliefs-Physical (FABQ-P)	ΔFear-avoidance beliefs-Work (FABQ-W)	ΔKinesiophobia (TSK)	ΔPattern of activity-Avoiding (POAM-A)	ΔPattern of activity-Overdoing (POAM-O)	Total
**Univariable analysis correlations**										
Catastrophism at entry (EnPCS)		**+**	**+**	**+**	**+**		**+**		**–**	**6**
Anxiety at entry (EnHAS)	**+**		**–**	**+**	**+**		**+**	**–**		**6**
Biomechanical disorders	**+**		**+**		**+**		**+**		**+**	**5**
BZD and Z-drugs	**+**		**+**				**+**	**+**		**4**
Non-specific back pain	**–**	**–**	**–**	**–**						**4**
Pain impact at entry (EnBPI-I)			**–**					**–**	**+**	**3**
Fear-avoidance beliefs work at entry (EnFABQ-W)	**+**	**+**			**+**					**3**
Peripheral osteoarthritis		**–**	**–**							**2**
Personality disorder				**–**			**–**			**2**
PTSD and EPCACE			**+**				**–**			**2**
Fear-avoidance beliefs Physical at entry (EnFABQ-P)			**–**					**+**		**2**
Pattern of activity-Avoiding at entry (EnPOAM-A)				**+**		**–**				**2**
Pattern of activity-Overdoing at entry (EnPOAM-O)		**+**		**–**						**2**
Kinesiophobia at entry (EnTSK)				**+**					**–**	**2**
**Multivariable analysis correlations**										
Non-specific back pain	**–**	**–**	**–**	**–**						**4**
Catastrophism at entry (EnPCS)			**+**				**+**		**–**	**3**
Biomechanical disorder	**+**		**+**		**+**					**3**
BZD and Z-drugs	**+**						**+**			**2**
Personality disorder				**–**			**–**			**2**
Fear-avoidance beliefs-Physical at entry (EnFABQ-P)			**–**					**+**		**2**
Anxiety at entry (EnHAS)					**+**			**–**		**2**

Only variables that were associated with variation in more than one score are shown. Negative associations are marked with a minus sign (‘–’) and positive association with a plus sign (‘+’), the opposite of the beta coefficient: a ‘–’ sign means having the property implicate in a worse prognosis for the outcome in question.

BPI: Brief Pain Inventory; BZD: benzodiazepines; EPCACE: enduring personality change after catastrophic experience; FABQ: Fear-Avoidance Beliefs Questionnaire; HAS and HDS: the Hospital Anxiety and Depression Scale; ODI: Oswestry Disability Index; PCS: Pain Catastrophizing Scale; POAM: Patterns of Activity Measures; PTSD: post-traumatic stress disorder; TSK: Tampa Scale of Kinesiophobia; Z-drugs: zolpidem and zopiclone.

Additionally, some variables influenced only one score variation. The following are the variables positively influencing only one score variation in multivariable analysis: alexithymia, kinesiophobia at entry (EnTSK), the use of myorelaxants, syndrome (PTSD) or/and EPCAC, sleep efficiency and trazodone.

The following are the variables negatively influencing one score variation in multivariable analysis: age, any back pain, cervical pain, DFA, DFA-conflict (existence of disputes about the right of receiving DFA), BPI-Interference at entry (EnBPI-I), gender (male), hypermobility syndromes, the use of opiates, post-traumatic stress peripheral osteoarthritis (OA), radiculopathy, tricyclic antidepressants (TCA) and time off work.

## Discussion

This cross-sectional study sought to identify clinical and demographic predictors of response to a multimodal pain management programme (MMP) in patients with refractory MCPS, aiming to improve tailored treatment approaches and enhance patient selection for these interventions. Our findings confirm that, while MMPs provide significant short-term benefits in key pain-related outcomes—such as pain severity, disability, kinesiophobia and catastrophizing—certain patient subgroups respond differently. Specifically, patients with non-specific low-back pain exhibited poorer outcomes, while those with high baseline catastrophizing demonstrated greater improvements, reinforcing the role of psychological interventions in pain management. Additionally, socioeconomic factors, such as ongoing disputes over DFA, negatively influenced treatment response, highlighting the broader impact of financial stress on CP management. These results underscore the complexity of treating refractory MCPS and emphasize the need for individualized, multidimensional treatment strategies to optimize outcomes in these patients.

### Key findings and implications

#### Improvement in pain and functional scores

Our results demonstrated statistically significant improvements immediately after the MMP in 9 out of 12 evaluated scores, including measures of pain impact (BPI-I), pain severity (BPI-S), disability (ODI), kinesiophobia (TSK) and catastrophizing (PCS). Our results align with a 2021 systematic review and meta-analysis on MMPs efficacy which demonstrated median size treatment effect in pain severity, physical, mental and social health (range = 0.38–1.94) [17]. Other studies found that refractory multi-treated patients like ours had poorer results in terms of depression, well-being and pain severity after MMP [18, 19].

In our sample, no significant changes were seen in scores for fear-avoidance beliefs about work (FABQ-W) and activity patterns of overdoing (POAM-O) and avoidance (POAM-A). This indicates that while the 2-week inpatient MMP effectively addresses certain aspects of CP, additional strategies or a longer duration may be required to better target fear-avoidance behaviours and maladaptive activity patterns. However, the significant improvement in POAM-Pacing suggests some progress in pain management strategies.

#### Clinical and demographic predictors of outcomes

Multivariate analysis identified key clinical and demographic factors influencing outcomes.

***Non-specific low-back pain.*** This factor had the most negative impact on four outcome measures, including pain severity (BPI-S), disability (ODI) and quality of life (BPI-I), indicating that patients with this condition may need more tailored interventions in MMPs.

***Catastrophizing at entry.*** This was the most influential factor in univariable analysis and the second in multivariable analysis, affecting symptom perception and resulting in higher baseline scores. The MMP successfully reduced catastrophizing, improving outcomes, particularly for pain severity and kinesiophobia. Interestingly, initial catastrophizing negatively impacted changes in POAM-Overdoing scores, likely because catastrophic patients are less inclined to engage in overdoing behaviours due to their anticipation of negative outcomes. Consequently, they start with lower POAM-O scores, limiting room for improvement.

***Biomechanical disorders***. Patients with these conditions improved more in pain severity, beliefs associated physical activity avoidance behaviours and catastrophizing. We defined biomechanical disorders as musculoskeletal disorders resulting from improper body biomechanics, repetitive motions or sustained exertions that place stress on the body. The variable was considered present when the final medical diagnosis appointed this pathophysiological mechanism as central. Typical examples included significant scoliosis, peripheral OA when associated with abnormal gait, and tendinitis associated with repetitive behaviours or postures. One possible explanation for the good prognosis of these patients is that the overload mechanism in question is more easily treated by the disciplines included in our MMP, as physiotherapy, chiropractic, osteopathy and occupational therapy. The amelioration of beliefs associated with physical activity avoidance behaviours and catastrophizing suggests that psychoeducation also played a role in these patients’ amelioration. Alternatively, these diagnoses are more likely to be used when there is less objective structural musculoskeletal damage to point out, thus indirectly selecting patients with more resources to achieve real changes within the programme.

***Psychiatric conditions.*** Psychiatric conditions, such as personality disorders, PTSD and anxiety, and the use of benzodiazepines and Z-drugs, significantly impacted multiple pain-related MMP outcomes. These findings underscore the necessity for comprehensive psychiatric assessment and intervention as part of the MMP. Integrating mental health support into the treatment plan for patients with CP could potentially enhance the overall effectiveness MMPs.

***Disability financial aid (DFA).*** Patients involved in disputes over DFA had less improvement in POAM-Overdoing scores, and those who had ever applied for DFA showed lower likelihood of pain severity reduction (BPI-S), despite the outcome of the petition. These findings underscore the impact of socioeconomic factors on CP management, emphasizing the need for patient support services to address these challenges.

***Pain impact at entry.*** This was associated in multivariable analysis with better prognosis for the overdoing behaviour (POAM-O). Overdoing is a maladaptive behaviour in which patient fails to respect their body limits and faces physical consequences. These patients often link their self-image to their work performance, resulting in both direct pain and indirect psychological suffering due to perceived failure. The MMP’s psychoeducation against overdoing directly reduces pain impact and indirectly alleviates psychological distress, thus helping patients manage both physical and emotional aspects of their condition more effectively.

***Pain characteristics.*** Specific pain traits (e.g. type and location) significantly influenced treatment responses. For instance, nociplastic pain was associated with different response patterns compared with other pain types. This suggests that pain characteristics should be considered when designing and implementing MMPs to ensure they meet the diverse needs of the patient population.

A 2023 systematic review by Liechti *et al.* analysed prognostic factors for health-related quality of life (HRQoL) in 6668 MCPS patients. The authors circumvented the differences in outcome measures by grouping HRQoL measuring tools into four categories: physical hrQoL, mental hrQoL, Fibromyalgia Impact Questionnaire (FIQ) and the 15-dimensional HRQoL measure (15D). In total, 49 different prognostic factors were identified, and the authors found a tendency of better outcome for patients with fewer pain sites, lower levels of negative cognitive behavioural factors and higher levels of positive cognitive behavioural factors [20]. Our findings align with this review, as cognitive-behavioural factors also significantly influenced outcomes in our analysis. In our case, five of the seven variables most associated with outcomes in multivariable analysis are directly associated with cognitive-behavioural characteristics (catastrophism, BZD and Z-drugs use, personality disorders, fear-avoidance beliefs over physical and anxiety at entry). However, unlike the review, the number of pain sites did not impact our results.

## Limitations

Firstly, our study assessed outcomes only from pre- to post-treatment, without a later follow-up. While we captured immediate changes, we currently lack data on the long-term sustainability of these effects. A medium-term follow-up study is in progress.

Secondly, our models explain only 7–15% of the variation in outcomes, indicating that important variables may have been excluded and some included variables may be covariates of more relevant factors.

Lastly, the cross-sectional design limits causal inference, and reliance on self-reported measures could introduce bias. Future longitudinal studies with larger samples and objective measures are needed for confirmation.

## Conclusion

This study highlights the complex interplay of clinical, psychological and socioeconomic factors in determining immediate treatment response in patients with refractory MCPS undergoing an MMP. While significant improvements were observed in key pain-related measures, certain subgroups, particularly those with non-specific low-back pain and psychiatric comorbidities, experienced poorer outcomes. In contrast, reductions in catastrophizing were associated with better pain management and functional improvements, underscoring the importance of targeted psychological interventions in these programmes.

From a clinical perspective, these findings emphasize the need for personalized treatment strategies in MMPs. Patients presenting with high catastrophizing tendencies may benefit from early psychological intervention, while those with non-specific low-back pain might require additional biomechanical assessments and tailored rehabilitation plans. Furthermore, socioeconomic challenges, such as disputes over DFA, should not be overlooked, as they may hinder treatment progress.

While this study provides valuable insights, its cross-sectional design limits causal inferences. A longitudinal study is currently underway to assess the long-term sustainability of these effects and refine patient selection criteria.

## Data Availability

Raw data are available at: https://doi.org/10.5281/zenodo.14912373.
